# Performance Evaluation of Hot Mix Asphalt Modified with Biomass-Based Waste Chestnut Shells as Filler Replacement

**DOI:** 10.3390/ma19030512

**Published:** 2026-01-27

**Authors:** Ceren Beyza İnce

**Affiliations:** Department of Civil Engineering, Faculty of Engineering and Natural Sciences, Malatya Turgut Ozal University, Malatya 44900, Turkey; ceren.ince@ozal.edu.tr; Tel.: +90-05078315591

**Keywords:** hot mix asphalt, chestnut shells, biomass, fatigue, creep stiffness, stability, sustainable pavement

## Abstract

**Highlights:**

**What are the main findings?**

- The stability of mixtures was increased by up to 10.3% with CNS additive.
- The resistance of pavements to moisture damage was improved with CNS additive.
- The resistance of pavements with CNS additive to rutting under moving loads increased by up to 249%.
- The fatigue life of pavements increased 3.81 times with CNS.

**What is the implication of the main findings?**

- It was observed that CNS additive can be used in road engineering and the optimum ratio is 10%.

**Abstract:**

This study aims to investigate the feasibility and performance effects of using waste chestnut shells (CNS), derived from agricultural biomass, as a filler replacement material in hot mix asphalt mixtures. The influence of CNS on the mechanical behavior of hot mix asphalt mixtures was evaluated through a comprehensive experimental program. Initially, the physical and conventional properties of the B50/70 asphalt binder, aggregates, and CNS material were characterized to establish a reference framework for mixture design. The optimum asphalt content (OAC) for the control mixture was established using the Marshall mix design procedure. Mixture specimens incorporating CNS were produced by introducing the material at four different proportions, corresponding to filler substitution levels ranging from 5% to 20% by weight. The prepared specimens were evaluated through a series of mechanical and durability-related tests, including Marshall stability and flow, Retained Marshall, moisture damage, dynamic creep stiffness, indirect tensile strength (ITS), fatigue performance, and indirect tensile stiffness modulus (ITSM). The results indicated that mixtures with 10% CNS replacement exhibited notable improvements in stability, water sensitivity, ITS, ITSM, dynamic creep, and fatigue resistance, suggesting that CNS has the potential to enhance the performance characteristics of hot mix asphalt pavements.

## 1. Introduction

Asphalt binders, which are viscoelastic and thermoplastic materials, are the most commonly used materials in road pavements. These materials play an important role in the structural integrity and durability of road pavements due to their ability to adhere to aggregates and their resistance to moisture. Aggregates, which are typically basic (limestone) or neutral (basalt) in structure, are road materials that have a significant effect on the stability and internal friction resistance of asphalt mixtures. For this reason, it is extremely important to know the properties of the asphalt binders and aggregates to be used in the mixture for the performance of hot mix asphalt (HMA) pavements [1,2,3].

HMA is obtained by mixing asphalt binder at a ratio of approximately 5–7% by weight with aggregate at a ratio of approximately 93–95% by weight at a specific temperature. HMA pavements are expected to be resistant to heavy vehicle loads, resistant to rutting, and capable of withstanding all types of weather/environmental conditions. However, due to various design and application errors, various deteriorations (such as rutting, cracking, settlement, undulation, and separation) occur in HMA pavements over time. These deteriorations in the pavement not only reduce the service life of the pavement but also significantly decrease its performance. Consequently, numerous modification strategies have been explored over time to enhance pavement performance and extend service life through the incorporation of various additives into asphalt binders or mixtures [4,5,6,7].

The most commonly used additives in asphalt or mixtures are polymers such as SBS, SBR, EVA, and Elvaloy Ret [8,9,10] as well as various vegetable and animal oils [11,12], nanomaterials [13,14], rubber [15,16], fibers [17,18] and waste plastics [19,20,21,22,23,24], which are used as modifying materials. Upon reviewing the studies conducted, it is evident that the majority of these additives only partially improve the properties of asphalt/mixtures and are not economically viable. Consequently, researchers have begun to use biomass-based materials in asphalt/mixtures as well [25,26].

Biomass refers to organic materials of plant, animal, agricultural, and industrial origin that are known as renewable energy sources used in various fields (engineering and materials, etc.), primarily for energy and fuel production. The vast majority of these materials are agricultural and industrial waste or by-products [27,28]. When looking at the biomass-based waste materials used in asphalt/mixtures, it has been stated that materials such as wood and by-products [29,30], oils [31], coffee grounds [32,33], animal manure [34], sunflower [35], corn [36,37], oats [38,39], and coconut [40] and walnut shells [41] significantly improve the properties of asphalt/mixtures. The use of chestnut shells as an additive in asphalt/mixtures has not yet been encountered in previous studies.

Several studies have investigated the use of lignocellulosic biomass materials and natural fibers as fillers or modifiers in asphalt mixtures. For instance, walnut shells and coconut shells have been evaluated as alternative fillers, demonstrating improvements in stiffness and rutting resistance due to their rigid particle structure and high lignin content [42,43,44]. Similarly, bamboo fibers have been used to reinforce the asphalt matrix, enhancing tensile strength and fatigue performance by acting as bridging elements across micro-cracks [45,46]. These materials generally improve the mechanical properties of asphalt mixtures through reinforcement, improved binder–aggregate interaction, and increased mastic stiffness. However, the specific effects of chestnut shells (CNS), which have a unique fibrous and porous structure, have not been previously investigated in asphalt mixtures. Compared to other agricultural residues, CNS are expected to provide additional benefits such as enhanced binder absorption, improved mechanical interlocking, and better stress distribution under repeated loading.

Chestnuts are a nutritious food that contributes to human nutrition. Chestnuts grow on chestnut trees and vary depending on the geography in which they grow. The natural distribution area of these trees is mainly the temperate regions of the Northern Hemisphere, and they are cultivated in China, Korea, Japan, Turkey, North America, Southern Europe, and Bolivia [47].

The chestnut tree is a tree with reddish-colored bark and hard leaves. There are 16 known species of chestnut trees today, which can live for approximately 500–1000 years. The most well-known of these are the European chestnut, American chestnut and Japanese chestnut. Considered an important tree species for agriculture and forestry economies, chestnuts have been one of the most important food sources in rural Europe for centuries. Chestnuts are usually consumed fresh, cooked, steamed, roasted, boiled or fried [47,48,49,50,51].

Global chestnut production was reported to be 2.3 million tonnes in 2017. This number was reported to be 2,095,741 tonnes in 2023. According to FAO data, 73% of this production belongs to China, 9% to Spain, 3.9% to Bolivia, and 3.6% to Turkey [47]. Approximately 77% of this chestnut production is exported to many countries, primarily Spain, Brazil, Italy, the United States (US), France, and Switzerland [51].

Chestnuts consist of two parts: the fruit and the shell (inner and outer). The shells account for 20% of the total weight of the fruit [51,52,53]. Chestnut shells (CNS) are a lignocellulosic biomass source with no commercial value. As an industrial–agricultural waste product, CNS are typically incinerated in factories for energy/fuel production or used in the wine aging process [51].

Despite the increasing interest in biomass-based additives for asphalt mixtures, there is a lack of research focusing on chestnut shells as a sustainable filler replacement. Therefore, this study aims to fill this gap by evaluating the feasibility of using waste chestnut shells as a partial filler replacement in hot mix asphalt. The main contributions of this study are (i) introducing CNS as a novel lignocellulosic filler in asphalt mixtures, (ii) investigating its effects on mechanical and durability-related properties such as moisture susceptibility, rutting resistance, and fatigue performance, and (iii) providing mechanistic insights into the observed performance changes through a comprehensive experimental program. The findings are expected to contribute to sustainable pavement design by utilizing agricultural waste material.

In this study, chestnut shells (CNS), which have not been previously applied in road engineering studies, were used as a partial replacement for filler material in hot mix asphalt in order to investigate their effects on mixture performance. For this purpose, the physical and volumetric properties of the asphalt binder and aggregates were first determined. The chemical and microstructural characteristics of the CNS additive were then identified using SEM, XRD, and FTIR analyses. A control mixture was designed using the Marshall mix design method to determine the optimum asphalt content (OAC). Based on the OAC, CNS were incorporated as a partial filler replacement at different ratios (5%, 10%, 15%, and 20%) to prepare modified mixtures. The mechanical and durability-related performance of all mixtures was evaluated through Marshall stability and flow, Retained Marshall, dynamic creep stiffness, indirect tensile strength (ITS), fatigue performance, indirect tensile strength ratio (ITSR), and indirect tensile stiffness modulus (ITSM) tests. The results demonstrated that CNS incorporation, particularly at an optimum replacement level, can improve the mechanical performance and moisture resistance of hot mix asphalt mixtures, highlighting the potential of CNS as a sustainable filler alternative in pavement applications.

## 2. Materials and Methods

### 2.1. Materials

B50/70 asphalt binder was obtained from Kırıkkale/Turkey Petroleum Refinery Inc. (TUPRAŞ) for use in the study. Table 1 summarizes the results obtained from the conventional binder tests.

**Figure 1:**
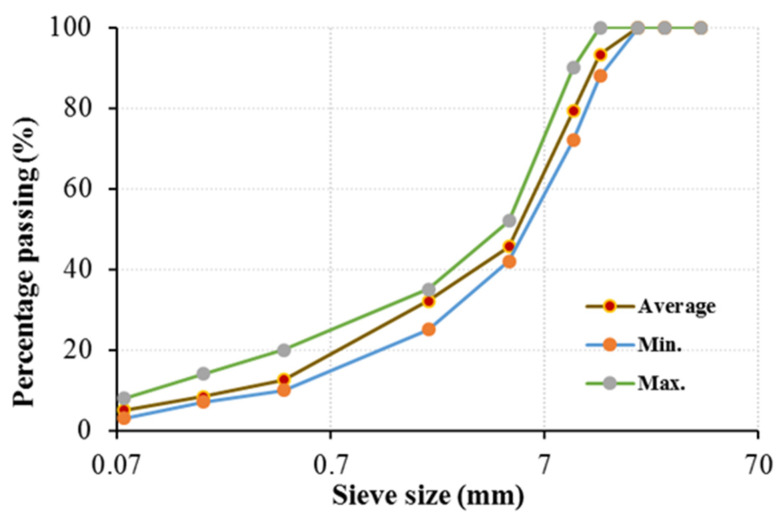
Gradation used.

The aggregate used in the study is limestone. The volumetric properties of the aggregates used in the mixture design are given in Table 2, and the gradation (with lower and upper limits) is given in Figure 1.

**Figure 2:**
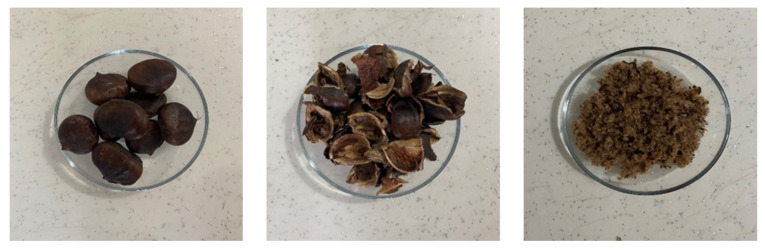
Chestnut shells (macroscopic view).

**Figure 3:**
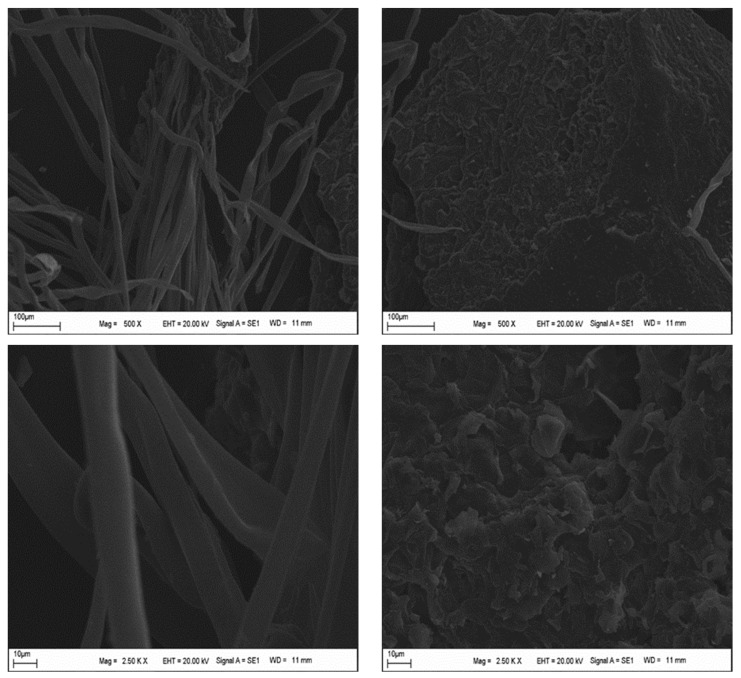
SEM images of chestnut shells.

**Figure 4:**
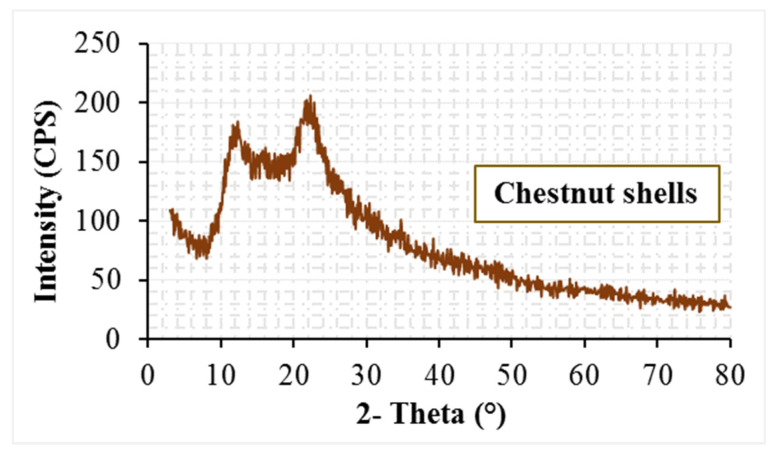
XRD pattern of chestnut shells.

**Figure 5:**
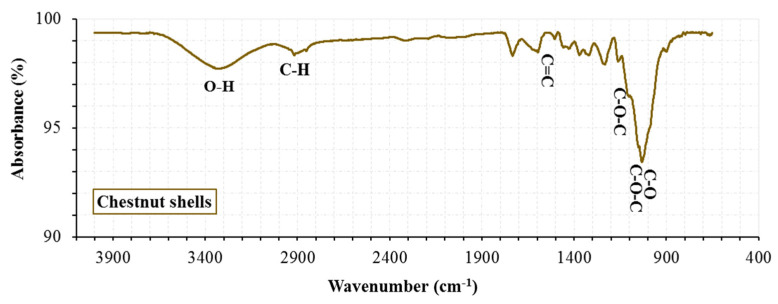
FTIR spectrum of the chestnut shell material with the main functional groups indicated.

The chestnut shells used in the mixture (Figure 2) were obtained by collecting the discarded shells of chestnuts whose fruit had been consumed. These waste materials were first dried at 100 °C and then ground. Table 3 summarizes the physical and chemical properties of the biomass-based CNS. Although waste chestnut shells exhibit a fibrous morphology, their particle size after grinding (<75 µm) enables them to behave as a filler-type material rather than a conventional fiber in the asphalt mixture. The SEM, XRD, and FT-IR analysis results for the waste chestnut shells are presented in Figure 3, Figure 4 and Figure 5.

SEM images reveal that the material has a generally fibrous and heterogeneous structure. Long, cylindrical cellulose fibers from the CNS additive are clearly visible, while the additive also has a rough, broken, and irregular surface morphology. This behavior is attributed to the coexistence of cellulose fibers and amorphous lignin–hemicellulose regions in the lignocellulosic structure of the CNS additive. The fact that the fibers are approximately 5–20 µm in size confirms the natural plant fiber character of the shell.

When examining the XRD pattern of the CNS additive, it is observed that the material predominantly exhibits an amorphous structure. The broad diffraction feature located around 6–10° (2θ) is associated with lignin, hemicellulose, and disordered cellulose regions, which are commonly observed in biomass-derived materials with a lignocellulosic structure. Another broad hump appearing approximately at 14–16° (2θ) corresponds to the amorphous shoulder region of cellulose type I. A weak and broadened diffraction feature related to cellulose type I can be observed around 22–23° (2θ), indicating a low degree of crystallinity. Overall, the XRD results suggest that the CNS additive is a lignocellulosic material dominated by amorphous phases, with a limited amount of semi-crystalline cellulose content.

When both results are considered together, it can be stated that the fractured and heterogeneous structures observed in the SEM image are consistent with the amorphous structure in the XRD pattern.

Upon examination of the IR spectrum, the lignocellulosic biomass structure of the chestnut shells is clearly visible. The stretching band corresponding to the wavelength and the presence of materials are shown in Table 4.

The high-intensity O-H stretching band at approximately 3400 cm^−1^ in Figure 5 is a fundamental characteristic of biomass, indicating the cellulose, hemicellulose, and lignin bonds in the structure. The C-H band at approximately 2900 cm^−1^ indicates the presence of polysaccharides and lignin, while the C-O and C-O-C bands at 1000–1160 cm^−1^ confirm the presence of a glycosidic skeleton. Furthermore, the C=O band at a wavelength of 1730 cm^−1^ indicates ester bonds due to hemicellulose. The presence of lignin in CNS is clearly seen at 1515 cm^−1^ with the aromatic ring C=C. The spectrum results show that the CNS contribution is an acidic biomass with a lignocellulosic structure containing active hydroxyl, carboxyl, and phenolic groups.

### 2.2. Method

**Figure 6:**
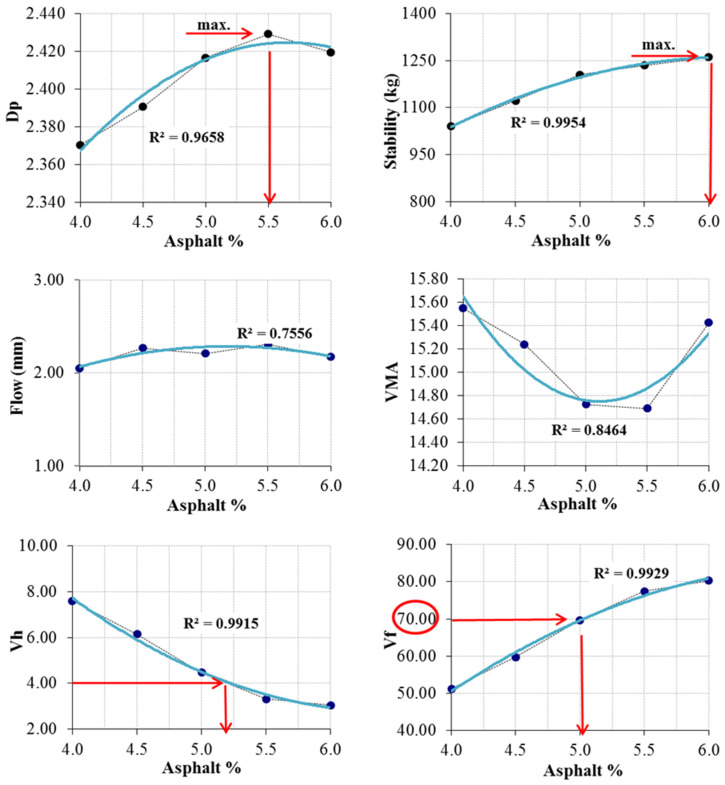
OAC determination graphs.

To evaluate the influence of waste chestnut shells on the performance of hot mix asphalt, reference (unmodified) mixture specimens were initially produced following the Marshall mix design procedure in accordance with ASTM D1559. The Marshall test determined OAC to be 5.40% (Figure 6).

**Figure 7:**
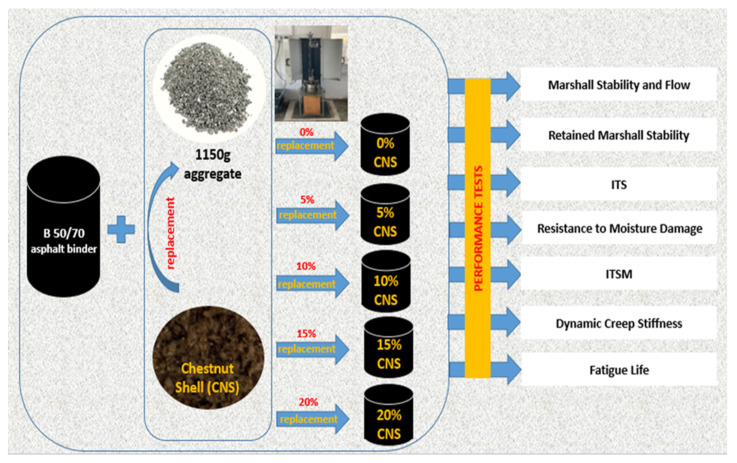
Experimental procedure flowchart.

Then, keeping the OAC constant at 5.40%, CNS-added mixture specimens were prepared by adding CNS at ratios of 5%, 10%, 15% and 20% by weight in place of the filler material in the mixture. In each specimen, the asphalt binder and 1150 g of aggregate were heated to approximately 160 °C. Each specimen was compacted by applying 75 hammer blows per side, yielding a total of 150 blows applied from a hammer drop height of 457 mm. All mixture specimens were prepared during the same process. The experimental procedure followed in the study is given in Figure 7.

The unmodified and CNS-replacement mixture specimen codes are presented in Table 5. The CNS content levels were determined based on preliminary trial mixtures and findings reported in the literature on biomass-based and lignocellulosic filler replacements in asphalt mixtures. The selected replacement ratios represent low, medium, and high CNS contents, enabling the evaluation of the effect of CNS incorporation on mixture performance without adversely affecting workability or volumetric properties. The upper limit of CNS content was defined considering practical limitations observed during trial mixing and compaction stages.

## 3. Experimental Program

### 3.1. Marshall Stability and Flow

The Marshall stability and flow values of all mixture specimens were determined using the Marshall test conducted in accordance with the ASTM D1559 standard. Both control and CNS-modified specimens were conditioned in a water bath at 60 °C for 30 min, followed by loading at a deformation rate of 50.8 mm/min. As a result of the test, the resistance of the specimen to pressure at maximum load is expressed as “stability”, and the vertical deformation at the moment the load reaches its maximum is expressed as “flow”. The Marshall ratio (MQ) parameter is obtained by dividing these two values. The Marshall Quotient (MQ) provides an effective measure of an asphalt mixture’s resistance to rutting and permanent deformation [54,55,56].

### 3.2. Retained Marshall

To determine the resistance of hot mixes to moisture damage, pure and CNS-modified specimens were subjected to the RMS test. For the test procedure, the specimens were classified into two groups. The conditioned group was stored in a water bath at 60 °C for 24 h, whereas the unconditioned group was maintained at the same temperature for approximately 40 min. They were then subjected to the Marshall test, and the RMS value was obtained by dividing the stability value of the conditioned specimen by the stability value of the unconditioned specimen [57].

### 3.3. ITS

Tensile strength characteristics of hot mix asphalt were evaluated using the ITS test in accordance with the ASTM D4123 specification. The test was performed using a Marshall apparatus at a load increase rate of 50 mm/minute. The ITS value was calculated using Equation (1). Here, “Pmax” represents the maximum applied load, “t” represents the thickness of the specimen, and “d” represents the diameter of the specimens [54].

(1) $$ITS=\frac{2Pmax}{\pi td}$$

In addition to tensile strength, the ITS test provides useful insight into the stiffness-related behavior and overall durability of asphalt mixtures [55].

### 3.4. Resistance to Moisture Damage

The moisture susceptibility of the hot mix asphalt was evaluated following the AASHTO T283 procedure. For testing purposes, the specimens were classified into two categories, namely, conditioned and unconditioned. Conditioned specimens underwent four stages. In the first stage, the voids in the specimens were filled with approximately 70% water using a vacuum. In the second stage, the specimens were kept at −18 °C for 16 h. In the third stage, the specimens were removed from −18 °C and were kept in a 60 °C curing bath for 24 h. In the fourth stage, the specimens were kept in a 25 °C water bath for another 2 h and subjected to crushing. The resulting measurement corresponds to the ITS value of the conditioned specimens (ITS_wet_). In contrast, the unconditioned specimens were immersed in a water bath at 25 °C for 2 h without prior conditioning and then tested to obtain the ITS value of the dry specimens (ITS_dry_). Following the completion of testing, ITSR was calculated using Equation (2). The ITSR values of hot mix specimens are expected to be greater than 80% [54,55].

ITSR = (ITS_wet_/ITS_dry_) × 100 (2)

### 3.5. ITSM

According to ASTM D4123 standard, the stiffness of asphalt mixtures is determined by the ITSM test. Stiffness is crucial in evaluating the dynamic load-carrying capacity of hot asphalt pavements. Thus, it provides insight into the pavement’s fatigue performance [34]. In this study, the ITSM test was applied under repeated loading with a haversine waveform. The stiffness modulus was calculated according to Equation (3). Here, “Sm” is the stiffness modulus (MPa), “P” is the repeated load (N), “t” is the thickness of the specimen (mm), “ΔH” is the recoverable horizontal deformation (mm), and “ν” is the Poisson’s (assumed to be 0.35) [54,58].

(3) $$Sm=\frac{P(v+0.27)}{t\Delta H}$$

ITSM test was conducted on all specimens at two test temperatures (10 °C and 20 °C) in accordance with the BS DD 213 standard. Each specimen was initially subjected to five conditioning load pulses, followed by five primary loading cycles to evaluate stiffness under dynamic conditions. The loading protocol employed a load period of 3000 ms, a Poisson’s ratio of 0.35, a target horizontal deformation of 5 μm, and a load rise time of 124 ms.

### 3.6. Dynamic Creep Stiffness

**Figure 8:**
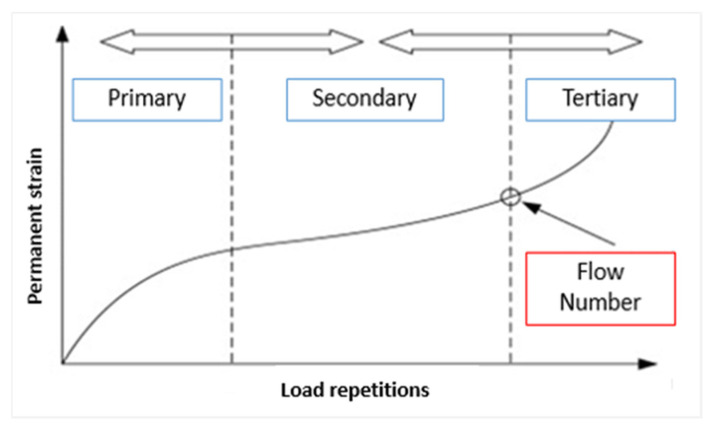
Flow number for the specimens [61].

Progressive shear deformation in hot mix asphalt layers leads to the development of rutting under repeated traffic loading. Therefore, pavements are expected to be resistant to shear deformation. The test is used to assess the shear deformation resistance of HMA. In the experiment, the deformation caused by repeated loading on a cylindrical specimen is measured. As a result of the deformation measurement, a dynamic creep graph of the specimen under repeated loading is plotted (Figure 8). In this graph, the beginning of region 3 is accepted as the flow number (FN), and this value is used to determine the resistance to rutting [59,60,61].

The dynamic creep test was performed in accordance with NCHRP 9–19 at a test temperature of 50 °C. Prior to loading, the specimens were conditioned under a preload of 10 kPa for 10 min. Subsequently, a semi-sinusoidal load was applied using a contact stress of 10 kPa and a deviator stress of 500 kPa. The test was terminated after 10,000 loading cycles, after which deformation–loading curves were generated for each specimen. The creep stiffness value was calculated at the conclusion of the test using Equation (4), where E_c_ (MPa) is the creep stiffness, σ (MPa) is the applied dynamic stress, and ε_c_ is the total residual strain [61].

E_c_ = σ/ε_c_ (4)

### 3.7. Fatigue

**Figure 9:**
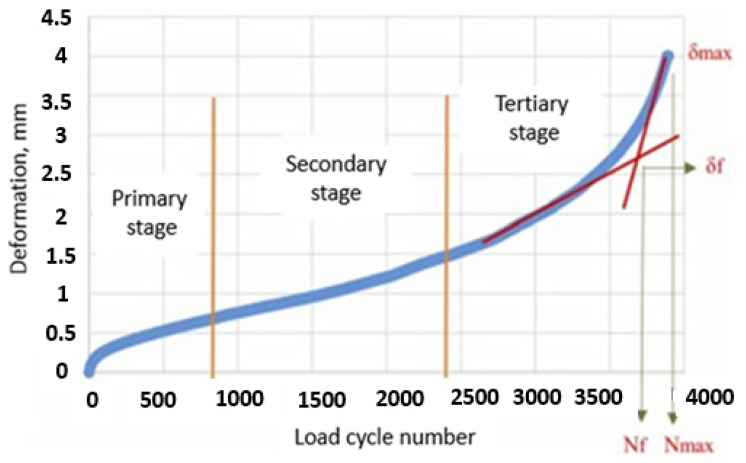
Load cycle number–deformation graph (representative).

Fatigue cracking directly affects the quality and service life of hot mix asphalt. In this study, specimens were subjected to fatigue testing in accordance with EN 12697–24. The test was continued until the specimens separated, and the fracture life was determined at the end of the test. A representative load cycle number–deformation graph based on the results of the stress-controlled test is shown in Figure 9 [4].

The experiment was conducted at 25 °C under a load of 400 kPa. In the experiment, the loading time was set to 0.1 s and the rest time to 0.4 s, and the load was applied in a sinusoidal manner. At the end of the experiment, the fatigue life Nf, the maximum load cycle count N_max_, the df corresponding to Nf, and the d_max_ corresponding to N_max_ were determined using the equations of the curves drawn from the tangents of Regions 2 and 3 [4].

Throughout the study, all tests were performed on at least three replicate specimens for each mixture type to ensure the repeatability and reliability of the experimental program. The average values are reported in the results, and the corresponding standard deviations were calculated and included where applicable. The relatively low standard deviations observed across the experimental results indicate good consistency and repeatability of the measurements. Furthermore, error bars representing data variability were added to the relevant figures to improve the statistical interpretation of the reported trends.

## 4. Results and Discussion

### 4.1. Marshall Stability and Flow

**Figure 10:**
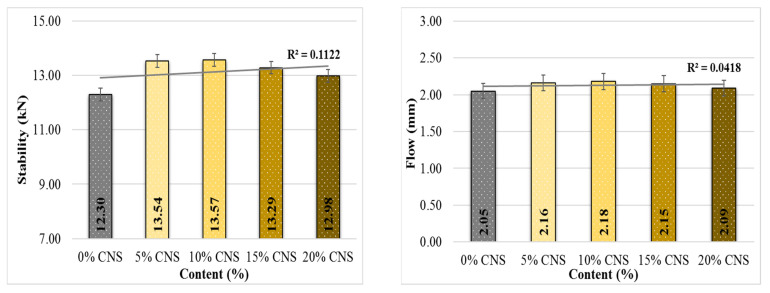
Stability and flow results according to CNS content.

**Figure 11:**
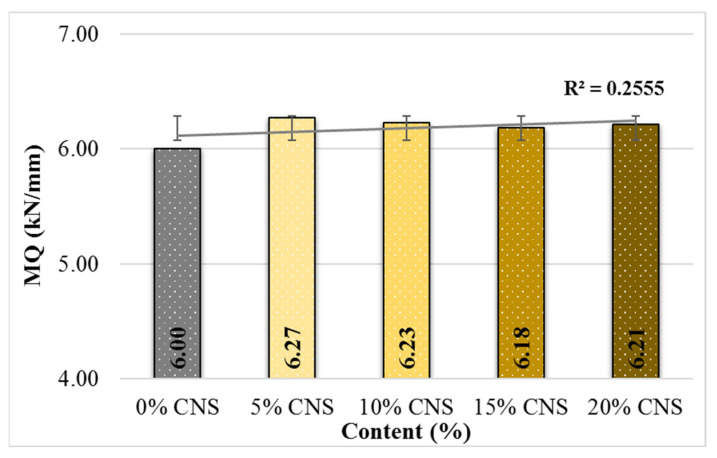
MQ values according to CNS content.

Figure 10 and Figure 11 summarize the Marshall stability and MQ results obtained for all mixture specimens.

Marshall stability values varied with CNS content and showed an overall increasing trend compared to the control mixture. Relative to the 0% CNS specimens, stability increased by 10.1%, 10.3%, 8.1%, and 5.5%, respectively, with the highest value obtained at 10% CNS replacement. This result indicates that mixtures incorporating 10% CNS may exhibit improved resistance to permanent deformation. The observed enhancement can be attributed to the effective interaction between the CNS additive, asphalt binder, and aggregate matrix. Flow values exhibited only slight increases of 0.5%, 0.6%, 0.49%, and 0.2% compared to the control mixture. Since flow reflects the flexible–plastic behavior of asphalt mixtures under load, these minor changes suggest that CNS incorporation does not significantly alter the deformation characteristics of the mixtures. Similar increases in stability have been reported for mixtures containing lignocellulosic fillers such as walnut shell and coconut shell, where improved stiffness and aggregate–binder interaction were attributed to the rigid particle structure and high lignin content [42,43,44].

As shown in Figure 11, the MQ values increased by up to 4.5% with CNS replacement, with the maximum improvement observed in mixtures containing 5% CNS. Given that MQ represents the resistance of hot mix asphalt to shear stresses, this finding suggests that mixtures with 5% CNS replacement demonstrate the most favorable shear resistance performance.

### 4.2. Retained Marshall Stability

**Figure 12:**
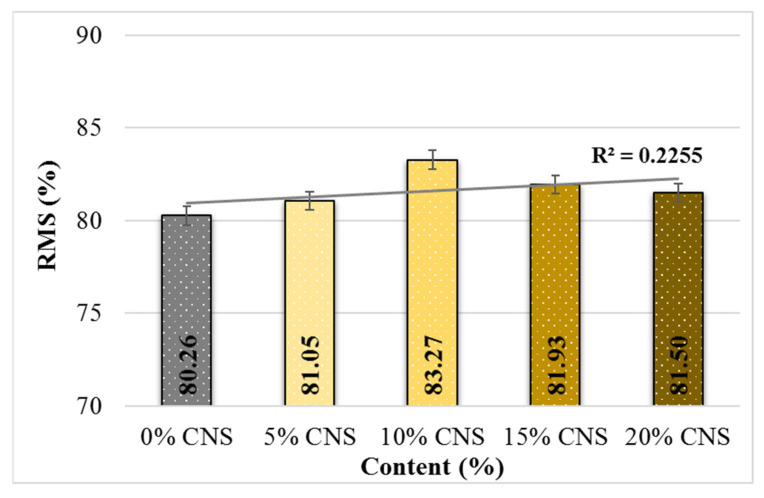
%RMS values according to CNS content.

The RMS test results for all mixture specimens according to CNS content are presented in Figure 12.

The results indicate that RMS values generally increase with CNS incorporation compared to the control mixture. Relative to the 0% CNS specimen, the RMS values increased by 0.01%, 3.8%, 2.1%, and 1.6%, respectively. This trend demonstrates that CNS addition enhances the moisture damage resistance of hot mix asphalt mixtures. Among the tested mixtures, the specimen containing 10% CNS replacement exhibited the highest RMS value, indicating superior resistance to moisture-induced deterioration. This improvement can be attributed to the enhanced adhesion between the asphalt binder, aggregates, and the CNS additive, which reduces the susceptibility of the mixtures to moisture damage.

### 4.3. ITS and ITSR

**Figure 13:**
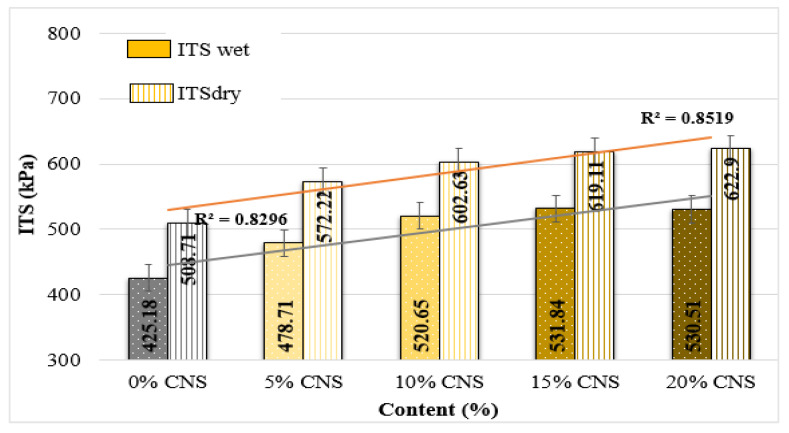
ITS values.

**Figure 14:**
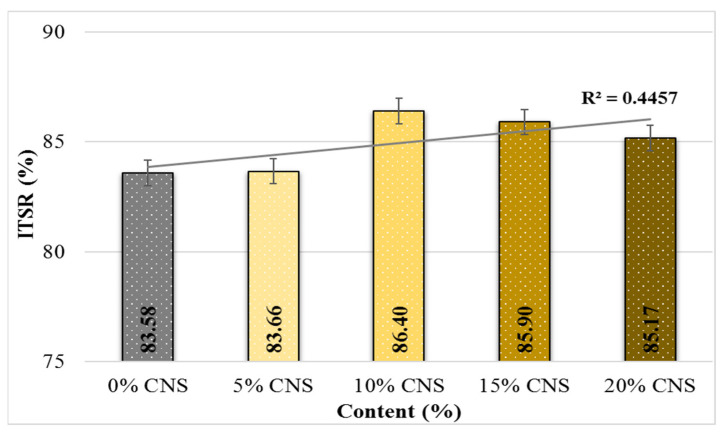
ITSR values of the specimens.

The effects of CNS incorporation on the ITS and ITSR of HMA mixtures are illustrated in Figure 13 and Figure 14.

Both wet and dry ITS values increased with CNS replacement compared to the control mixture, as shown in Figure 13. For moisture-conditioned specimens, ITS_wet_ values increased by 12.6%, 22.5%, 25.1%, and 24.8%, respectively, with increasing CNS content. Similarly, ITS_dry_ values exhibited increases of 12.5%, 18.5%, 21.7%, and 22.5% relative to the 0% CNS mixture. These improvements indicate that CNS incorporation enhances the tensile strength of asphalt mixtures under both dry and moisture-conditioned states. The observed increase in ITS values can be attributed to the improved adhesion between the CNS additive, asphalt binder, and aggregates. Considering the acidic nature of the CNS material and the asphalt binder, stronger interfacial bonding is expected, which contributes to higher tensile strength value. Similar increases in ITS values have been reported for mixtures containing coconut shell powder, which were attributed to improved binder–aggregate adhesion and increased stiffness due to the porous and lignin-rich structure of the additive [43].

Additionally, when examining the ITSR values of the CNS-replacement mixture specimens, it is observed that the ITSR value generally increases as the replacement ratio increases (up to a maximum of 3.4%). The ITSR value indicates the moisture sensitivity of hot mixes. According to the specification, it is expected to be higher than 80% [62]. Based on these results, it can be stated that the 10% CNS-replacement specimen has the most favorable result in terms of moisture sensitivity.

The observed improvement in moisture resistance may be related to the physical and chemical characteristics of CNS. CNS particles, due to their porous and polar surface characteristics, can enhance the adhesion between asphalt binder and aggregates. The micro-roughness of CNS provides additional mechanical interlocking, while the lignin-rich surface may promote better compatibility with the asphalt binder through physical adsorption. This improved binder–aggregate interaction reduces moisture susceptibility and contributes to higher ITS and ITSR values.

### 4.4. ITSM

**Figure 15:**
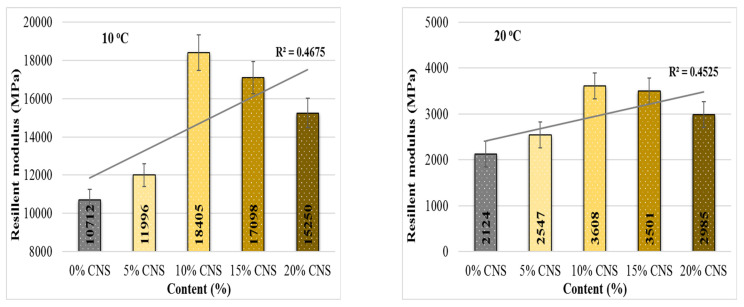
ITSM results at two different temperatures.

The ITSM results obtained from all specimens tested at 10 °C and 20 °C are presented in Figure 15.

When examining the ITSM test results conducted at two different temperatures (Figure 15), it is observed that, in general, as the ratio increases compared to the mixture containing 0% CNS, the ITSM values also increase. At 10 °C, these increases are 20%, 71.8%, 59.6% and 42.4%, respectively; at 20 °C, they are 19.9%, 69.9%, 64.8% and 40.5%, respectively. The increase in the elasticity modulus of the mixture indicates an increase in resistance to permanent deformation. When the results are evaluated, it can be stated that the highest resistance to permanent deformation at both temperatures was obtained from mixtures with 10% CNS replacement. This behavior may be attributed to the lignocellulosic structure of the CNS additive, which may contribute to the improved resistance of the pavement to cracking under load.

### 4.5. Dynamic Creep Stiffness

**Figure 16:**
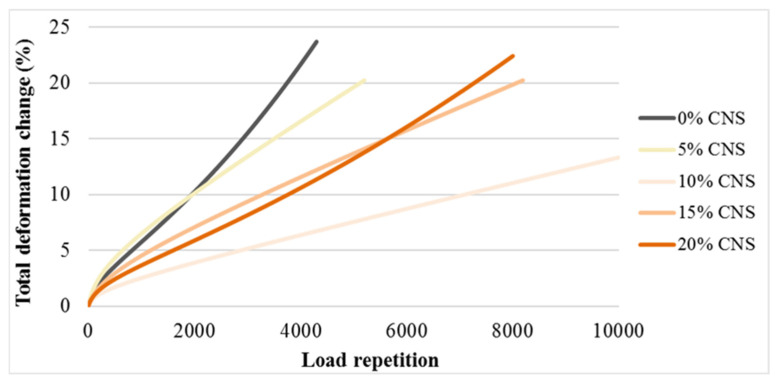
Load repetition–total deformation change curves for the specimens.

The load cycle number–deformation curves drawn as a result of the dynamic creep test applied under stress control on all pure and CNS-replacement specimens are presented in Figure 16.

**Figure 17:**
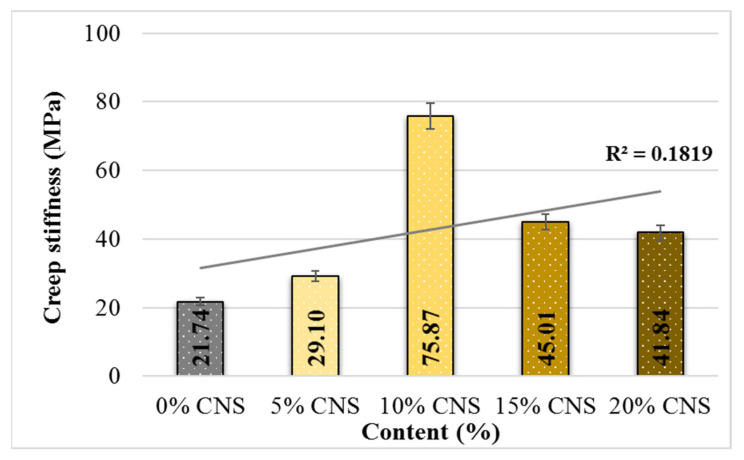
Change in creep stiffness values depending on CNS content.

Upon examination of the curves, it is observed that deformation generally increases linearly in all specimens. As the number of load cycles increased, all mixtures, except the one containing 5% CNS, displayed a degradation pattern comparable to that of the control mixture, up to approximately 2000 load cycles. The control specimen ultimately failed after 4200 load repetitions. For comparative purposes, creep stiffness values corresponding to 4200 load cycles were considered for all specimens. The relationship between CNS content and creep stiffness is presented in Figure 17.

An overall increasing trend in creep stiffness is evident as the CNS content in the mixtures increases. The increase rates are 33.9%, 249%, 107% and 93%. From these results, it can be stated that the specimens with 10% CNS replacement have the highest creep stiffness values and, therefore, these specimens have a higher permanent deformation resistance under repeated loads.

**Figure 18:**
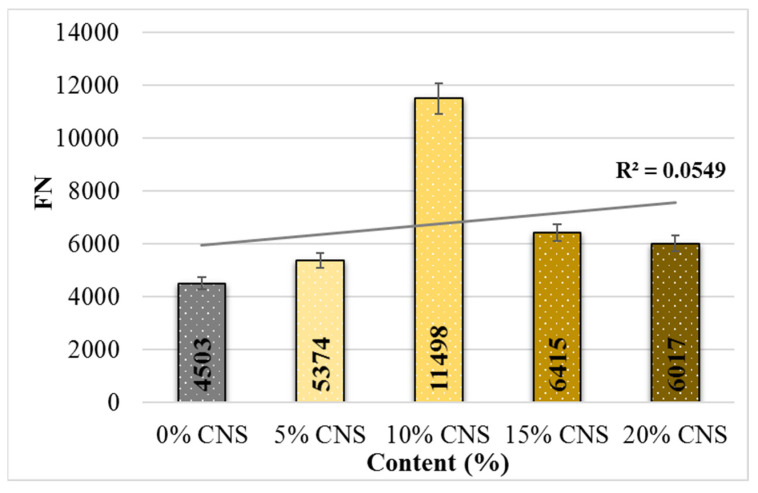
FN values for the specimens.

The equations used to calculate the FN values are given in Table 6, and the change in FN values according to CNS content is shown in Figure 18.

FN is a parameter representing the rutting resistance of HMAs and is obtained from the load repetition–total deformation curve. The FN values of the mixtures were determined by applying a second-order parabolic regression equation to the curve [62].

The FN values increased with CNS incorporation compared to the control mixture. Relative to the 0% CNS specimen, the increases were 19.3%, 155%, 43%, and 34%, respectively. Among the tested mixtures, the specimen containing 10% CNS replacement exhibited the highest FN value, indicating superior resistance to rutting under repeated loading. This improvement can be attributed to the lignocellulosic structure of the CNS additive, which enhances the resistance of hot mix asphalt to mechanical deterioration.

### 4.6. Fatigue

**Figure 19:**
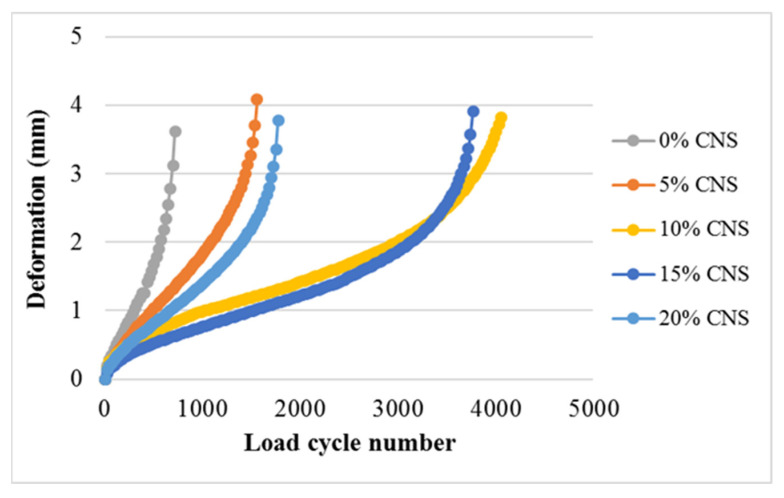
Load cycle number–deformation curves for the specimens.

Stress-controlled indirect tensile fatigue tests were conducted on all specimens, resulting in load cycle–deformation curves. As shown in Figure 19, the specimen with 0% CNS additive fractured with the lowest number of load cycles, while the specimen with 10% CNS additive required the highest number of load cycles to fracture. Additionally, it can be noted that the load cycle counts for the 10% and 15% CNS-added specimens were comparable. Ultimately, it is observed that more load cycles are required for specimens with CNS addition to fracture.

**Figure 20:**
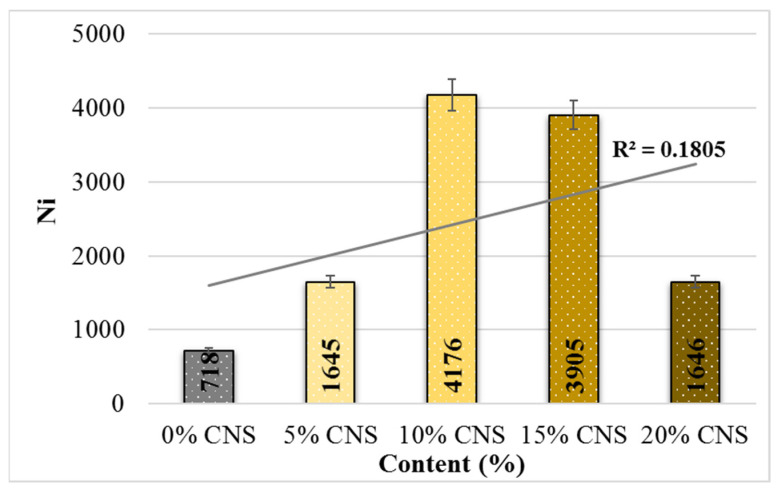
Relationship between Ni and the load cycles of the binders.

**Figure 21:**
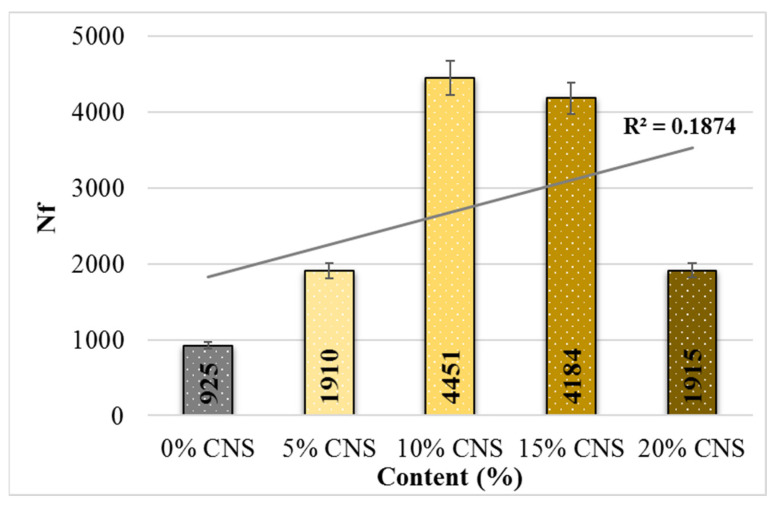
Relationship between load cycles and mixture with Nf.

Figure 20 shows the variance in the average load repetition number (Ni) for crack initiation in specimens with CNS additives at a stress level of 400 kPa. Figure 21 shows the average fatigue life (Nf) values of the specimens according to the CNS additive ratio. From this, it can be seen that with an increase in the CNS additive ratio, the average load cycle number (Ni) for crack initiation increased by 4.82 times compared to the unmodified mixture, and the fatigue life also increased by 3.81 times compared to the unmodified mixture (0% CNS). All these results were obtained from mixtures with 10% CNS additive.

**Figure 22:**
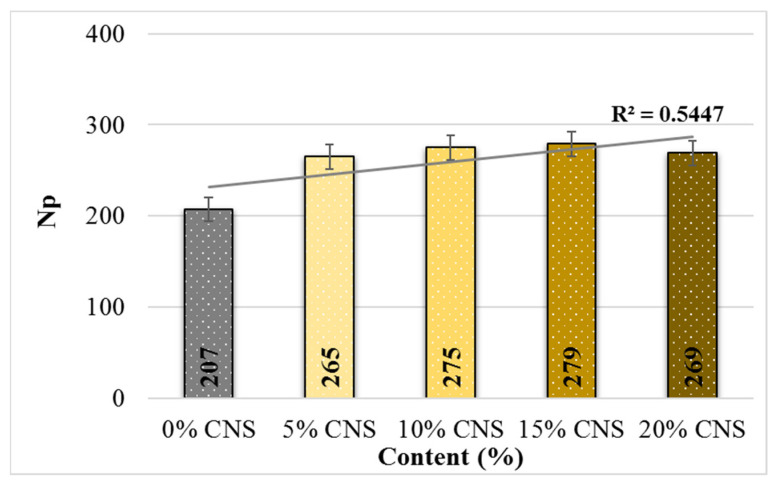
Relationship between Np and the load cycle counts of the mixture.

**Figure 23:**
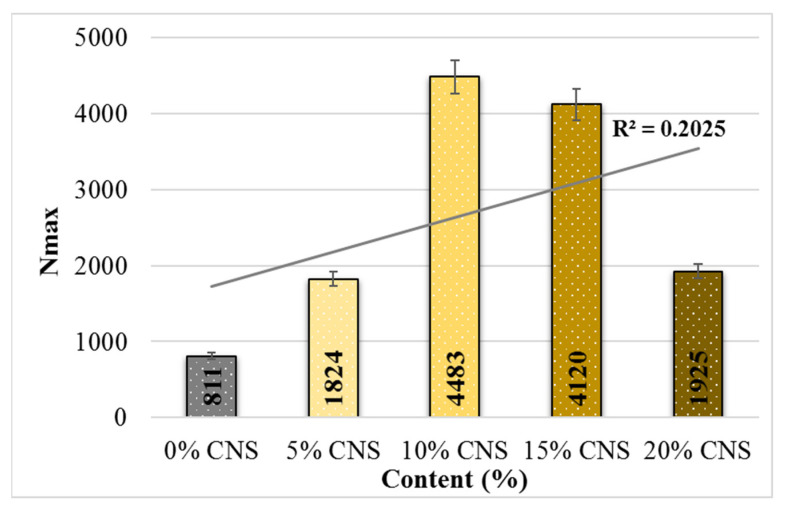
Relationship between Nmax and the number of load cycles of the mixture.

Figure 22 shows the crack propagation in the specimens with an increase in the CNS contribution ratio and the number of load cycles (Np), while Figure 23 shows the changes in the maximum number of load cycles (Nmax). From this, it can be seen that the lowest Np value was obtained from specimens with 0% CNS contribution, while the highest Np value was obtained from specimens with 15% CNS contribution. Looking at the Nmax values, it is seen that the value increases with CNS content, and the highest increase, up to 4.53 times, was obtained from mixtures with 10% CNS content.

When the indirect tensile fatigue test results are evaluated overall, it can be stated that the CNS additive improves the fatigue performance of hot mixes, with the most effective mix ratio being 10% CNS. The improvements in fatigue life and rutting resistance can be attributed to the lignocellulosic and fibrous structure of CNS. The cellulose and lignin components provide a reinforcing network within the asphalt matrix, enhancing stress distribution and energy dissipation under cyclic loading. This fibrous network acts similarly to short fibers in composite materials, limiting crack initiation and propagation by bridging micro-cracks and reducing strain concentration at weak points. Consequently, mixtures with CNS addition exhibit delayed fatigue damage and higher resistance to permanent deformation under repeated loading.

## 5. Conclusions

In this study, the effect of waste chestnut shells (CNS), a biomass-based material, on the performance of hot mix asphalt mixtures was investigated. The main findings of the study can be summarized as follows:

- The Marshall stability results indicated that CNS incorporation increased mixture stability, with the highest improvement observed in mixtures containing 10% CNS replacement. This suggests that CNS addition contributes to increased stiffness of HMA pavements.
- The Marshall Quotient (MQ) values increased with CNS addition, particularly at the 10% replacement level, indicating improved resistance of HMA mixtures to shear stresses.
- Retained Marshall Stability (RMS) results showed enhanced moisture damage resistance for mixtures with CNS replacement, with the most notable improvement observed at 10% CNS content.
- The ITS and ITSR results demonstrated that CNS-modified mixtures exhibited improved moisture sensitivity resistance and adhesion characteristics compared to the control mixture.
- The ITSM test results showed higher stiffness values for CNS-modified mixtures, especially at the 10% replacement level, indicating improved load distribution capacity.
- Dynamic creep test results revealed that CNS addition, particularly at 10% replacement, significantly improved resistance to permanent deformation and rutting under repeated loading.
- Fatigue test results indicated that the fatigue life of CNS-modified mixtures increased compared to the control mixture, with the highest performance achieved at the 10% CNS replacement level.

Overall, the results demonstrate that waste chestnut shells can be effectively used as a partial filler replacement in hot mix asphalt mixtures to enhance mechanical performance. In addition to performance improvements, the utilization of CNS contributes to environmental sustainability by enabling the recycling of agricultural waste materials. Future studies should focus on long-term field performance, aging behavior, and the durability of CNS-modified mixtures under different traffic and climatic conditions.

## Figures and Tables

**Table 1:** Characteristic properties of the binder.

Properties	Standard	Result
Penetration (0.1 mm)	ASTM D5	55
Softening point (°C)	ASTM D36	51
Specific gravity (g/cm^3^)	ASTM D70	1.033
Flash point (°C)	ASTM D92	324
Mixing temperature (°C)	-	160
Compaction temperature (°C)	-	147

**Table 2:** Physical and volumetric properties of aggregates.

Properties	Standard	Result
Coarse apparent specific gravity (g/cm^3^)	ASTM C127	2.752
Coarse volume specific gravity(g/cm^3^)	ASTM C127	2.700
Fine apparent specific gravity(g/cm^3^)	ASTM C128	2.688
Fine volume specific gravity(g/cm^3^)	ASTM C128	2.763
Filler apparent specific gravity(g/cm^3^)	ASTM D854	2.778

**Table 3:** Various physical and chemical properties of the biomass-based CNS additive.

Properties	Value
Material type	Biomass-based agricultural waste
Main constituents	Lignocellulosic, Cellulose, Lignin
Apparent density (g/cm^3^)	1.32
Water absorption (%)	10
Function in mixture	Filler-type material

**Table 4:** Wavelength and functional group.

Wavelength Range (cm^−1^)	Functional Group
3200–3600	O-H Band
2850–2950	C-H Band
1510–1515	C=C Band
1150–1160	C−O−C
1000–1100	C−O and C−O−C

**Table 5:** Specimens coding.

Content	Coded
B 50/70 asphalt binder + 1150 g aggregate (0% CNS replacement)	0% CNS
B 50/70 asphalt binder + 1150 g aggregate (5% CNS replacement)	5% CNS
B 50/70 asphalt binder + 1150 g aggregate (10% CNS replacement)	10% CNS
B 50/70 asphalt binder + 1150 g aggregate (15% CNS replacement)	15% CNS
B 50/70 asphalt binder + 1150 g aggregate (20% CNS replacement)	20% CNS

**Table 6:** Equations obtained from the curves of the specimens, R^2^ and FN values.

Specimens	Equation	R^2^	FN
0% CNS	y = −0.0051x^2^ + 45.928x + 10084	0.995	4503
5% CNS	y = −0.0035x^2^ + 37.620x + 15793	0.992	5374
10% CNS	y = −0.0026x^2^ + 59.794x + 18471	0.996	11,498
15% CNS	y = −0.0022x^2^ + 28.226x + 10541	0.995	6415
20% CNS	y = −0.0012x^2^ + 14.440x + 13251	0.997	6017

## Data Availability

The original contributions presented in this study are included in the article. Further inquiries can be directed to the corresponding author.
